# Foren-STR: a comprehensive tool for rapid calculation of forensic parameters from autosomal STR data

**DOI:** 10.1007/s00414-025-03540-z

**Published:** 2025-06-19

**Authors:** Jorge Castillo-Ortiz, Lizzette Salinas-Pineda, N. Sofía Huerta-Pacheco, Mariano Guardado-Estrada

**Affiliations:** 1 Laboratorio de Antropología y Odontología Forense, Escuela Nacional de Ciencias Forenses, Universidad Nacional Autónoma de México, Ciudad de México, México; 2 Laboratorio de Genética, Escuela Nacional de Ciencias Forenses, Universidad Nacional Autónoma de México, Ciudad de México, México; 3 SECIHTI, Ciudad de México, México

## Abstract

In forensic genetics, prior to solving paternity or identification cases, it is mandatory to analyze population data of genetic markers to ensure the quality of the estimations in the required calculations. Currently, there are several tools that analyze forensic population parameters and population genetic statistics. However, the use of some of these tools can be time-consuming, as there is a learning curve to get familiarized with them, a situation that can be complicated due to the workload of forensic laboratories. Here, we propose a comprehensive tool for the quick calculation of forensic parameters and some population genetic statistics for an autosomal Short Tandem Repeats (STR) database. This tool is a web-based application (https://enacif.shinyapps.io/Foren-STR/) developed in R, which estimates Hardy-Weinberg equilibrium (HW), probability of match (PM), power of discrimination (PD), and power of exclusion (PE). Additionally, it can analyze more than one population and graphically infer whether there is population substructure through a heatmap or by statistically calculating genetic distances, such as F_st_. The results of this tool are similar to those of other tools, providing an alternative and user-friendly option for forensic geneticists.

**Supplementary Information:**

The online version contains supplementary material available at 10.1007/s00414-025-03540-z.

## Background

Population genetics is a crucial tool in forensic genetics, providing the statistical support for genetic analyses in human identification and kinship testing [1]. To ensure reliable estimations in these analyses, forensic genetic laboratories employ population databases comprising various genetic markers, such as autosomal STRs [2]. These databases must represent individuals from the geographic region where the genetic tests are conducted and should be regularly updated to maintain quality standards in forensic genetics procedures [2].

The analysis of genotype data from such databases requires the calculation of forensic parameters. Among these, the allele frequencies of each autosomal STR marker is critical for subsequent analyses [4, 5]. Additionally, the Hardy-Weinberg equilibrium for each locus must be evaluated to identify potential deviations in the analyzed population [3]. Other important parameters include observed Heterozygosity (H), Probability of Match (PM), Power of Discrimination (PD), and Paternity Index (PI), among others [4]. These calculations are essential for assessing the utility and validity of population databases in forensic applications.

On the other hand, despite the availability of autosomal STR population databases for various geographical regions, they must be frequently updated due to the development of new commercial kits [5]. Each time a laboratory adopts a new kit, it is advisable to construct a population database specific to that kit. While numerous bioinformatic tools are available to perform these calculations, many require overcoming a learning curve, and the high workload in forensic laboratories often limits the time allocated for such analyses [6–8].

Here we introduce the Foren-STR, a bioinformatic tool designed to streamline the calculation of forensic parameters for autosomal STR markers. Foren-STR enables the estimation of key parameters, including allele frequencies, Hardy-Weinberg equilibrium, PM, and PD, among others. Additionally, it facilitates the comparison of genetic distances as an alternative to F_st_ values, providing a rapid means to infer population substructure among analyzed groups [9]. Foren-STR offers a user-friendly and efficient solution to meet the demands of forensic laboratories, ensuring the continued reliability and applicability of population databases in forensic genetics.

## Environment of Foren-STR

Foren-STR is an online tool based on R [10] and designed for calculating forensic parameters. This tool is a web application built using the Shiny framework [11] and can be accessed at the following URL: https://enacif.shinyapps.io/Foren-STR/. The Foren-STR has the option to translate from English to Spanish, the commands so it is useful for those users who are not familiarized with the English terms.

### Data input

Foren-STR accepts tab-delimited text tables. The first column must contain the sample ID, and the second column must contain the population ID. The following columns correspond to the STR data, with each locus requiring two columns. Foren-STR accepts point alleles and only analyzes diploid data. Once the data is uploaded, it can be visualized in the Description > Data Set tab. Additionally, in the input file section, users can select all loci or specific loci and populations for analysis. This allows users to perform the analysis on all populations and loci or to focus on selected subsets. Foren-STR’s input file format is similar to that of the STRAF software [7, 8], making them compatible.

## Calculations

### Allelic and genotype frequencies table

Foren-STR calculates both the allele and genotype frequencies for all loci from the input file and displays them in a table. These results can be viewed in the Description > Allelic Frequencies and Description > Genotypic Frequencies tabs. The allelic frequencies table is presented in a standard format and can be downloaded as a .TXT file or copy to clipboard. Similarly, the frequencies of all genotype combinations observed in the input file for all loci are displayed in a table and can also be downloaded same way.

**Fig. 1:**
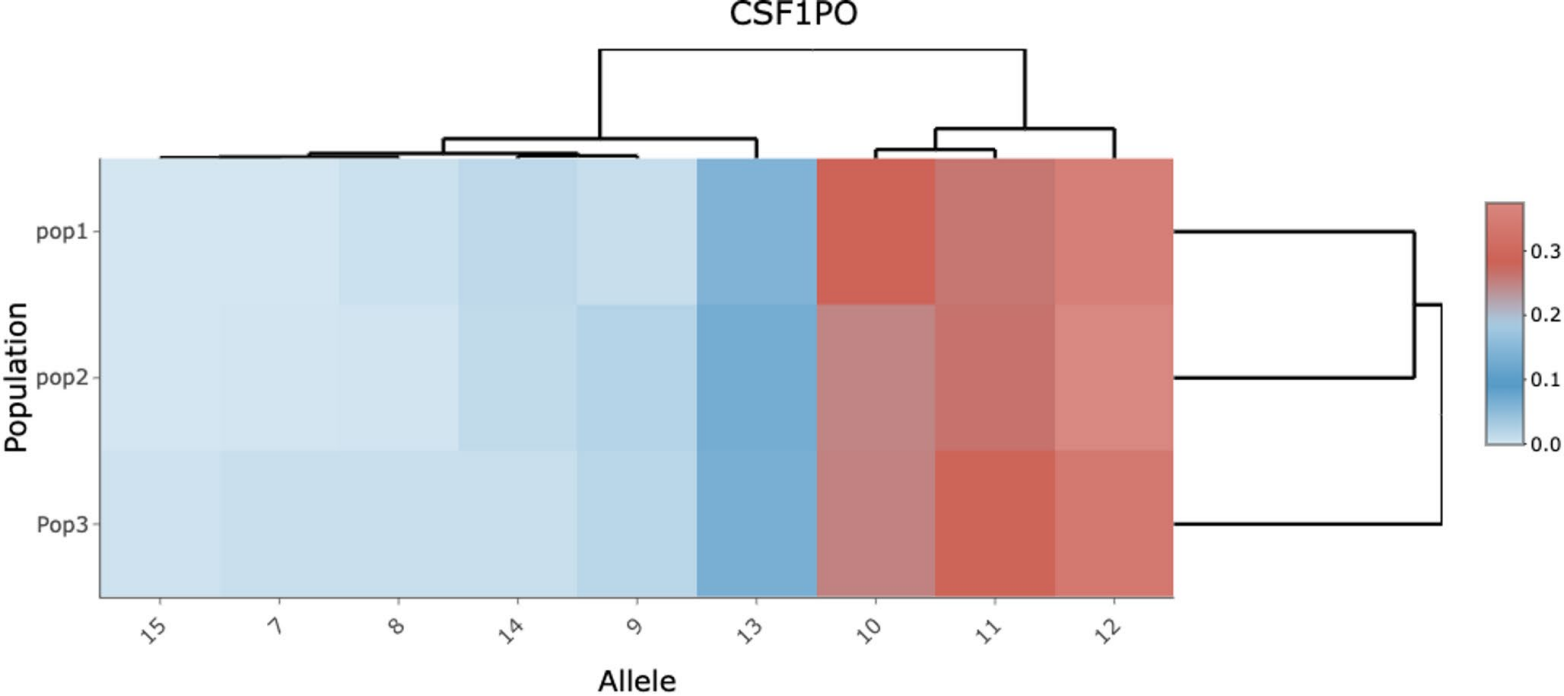
Heatmap of allelic frequencies among three populations. A heatmap of the allelic frequencies of CSF1PO among three populations is plotted. The color depicts the allele frequency from 0 (blue) to 0.3 (red). In the *y*-axis is the name of the population and in the *x*-axis the alleles found in that marker. According to allele frequencies, population 1 and population 2 are more closely related than population 3

To infer population substructure among different populations (Description > Heatmap tab), a heatmap based on the allelic frequencies of each locus in each subpopulation can be generated (see Fig. 1). This allows users to identify differences in allelic frequencies among subpopulations across all analyzed loci.

### Forensic parameters

This software enables the calculation of common forensic parameters using an STR population database [4, 7]. The forensic parameters estimated by Foren-STR include Hardy-Weinberg equilibrium (HW), expected heterozygosity (He), and observed heterozygosity (Ho). Other calculations include the combined paternity index (PIC), typical paternity index (ITP), power of discrimination (PD), probability of exclusion (PE), and probability of match (PM). These calculations are performed per locus and combined, except for HW and PIC. The individual or combined results of these parameters can be presented by subpopulation or for the entire dataset in a table (Table statistics > Statistics tab), which can be downloaded as a TXT file or copy to clipboard.

#### Hardy Weinberg equilibrium

The Foren-STR software estimates whether the analyzed population is in Hardy-Weinberg (HW) equilibrium [6, 12]. This test is essential when calculating forensic parameters for an autosomal STR population database, as it identifies potential population stratification in the analyzed population. Foren-STR employs the same HW calculation method as the Arlequin software and in the pegas R package, using the following formula:

$$\:HW=\frac{n!\prod\:_{i=1}^{k}{n}_{i}*!}{\left(2n\right)!\prod\:_{i=1}^{k}\prod\:_{j=1}^{i}{n}_{ij}!}2H$$

Where *n!* is the factorial of the sample size, *k* is the number of different alleles in the analyzed locus, *n*_*i*_​ is the allele frequency of allele *i*, *n*_*ij*_ is the number of individuals with genotype *i*, *j*, *2n* is the total number of alleles, and *H* is the frequency of heterozygotes for those alleles. This formula calculates the probabilities of all genotype combinations in the analyzed population. Using Markov-chain permutations and different contingency tables, the *p-value* of this calculation is estimated, allowing users to adjust for multiple comparisons, such as with Bonferroni’s correction.

#### Observed and expected heterozygosity

The observed heterozygosity (Ho) accounts for the proportion of heterozygous loci in individuals from a population. It is estimated by dividing the number of heterozygous individuals by the total number of individuals. On the other hand, the expected heterozygosity (He) is the probability that a locus is heterozygous and is calculated using the following formula:

$$\:He=\left[{\sum\:}_{j=1}^{n}\left(1-{\sum\:}_{i=1}^{m}{p}_{ij}^{2}\right)\right]/n$$

where *n* is the number of loci, *m* is the number of alleles found in locus *j*, and *pij* is the frequency of allele *i* in locus *j*.

#### Polymorphism information content

Similar to heterozygosity, the polymorphism information content (PIC) measures the level of polymorphism present in a genetic marker. It is calculated using the following formula:

$$\:PIC=1-{\sum\:}_{i=1}^{n}{p}_{i}^{2}-\left({\sum\:}_{i=1}^{n-1}{\sum\:}_{j=i+1}^{n}{2p}_{i}^{2}{p}_{j}^{2}\right)$$

where *n* is the number of alleles, and *pi* and *pj* are the frequencies of alleles *i* and *j*. As the PIC value approaches 1, the genetic marker becomes more effective at discriminating between two unrelated individuals in a population.

#### Probability of match

The probability of match (PM) is a common forensic parameter that estimates the probability of finding two identical genotypes in a population. It is calculated using the following formula:

$$\:PM={\sum\:}_{i}^{}{{(p}_{i})}^{2}$$

Where *pi* is the frequency of genotype *i* at a specific locus in a population.

#### Power of discrimination

The power of discrimination (PD) is the probability that two unrelated individuals can be genetically distinguished by analyzing one or several genetic markers. It is calculated as follows:

$$\:PD=1-PM$$

#### Power of exclusion

The power of exclusion (PE) is the fraction of individuals who have a different DNA profile from a randomly selected individual in a paternity case. It is estimated using the following formula:

$$\:PE={h}^{2}\left(1-2h{H}^{2}\right)$$

where *h* is the number of heterozygous individuals, and *H* is the proportion of homozygous individuals.

#### Typical paternity index

The typical paternity index (ITP) estimates how many times more likely it is that the analyzed individual is the biological father compared to a random individual. This calculation is performed per locus using the following formula:

$$\:TPI=\frac{1}{2H}$$

where *H* is the proportion of homozygous individuals.

#### Combined values

Two different approaches are used to estimate the combined values of forensic parameters. The combined values of Ho, He, PD, PM, and ITP are calculated by multiplying the individual values estimated for each marker. On the other hand, the combined values of PE and PD are calculated using the following formulas:

$$\:{PE}_{c}=1-\left(1-{PE}_{1}\right)\left(1-{PE}_{2}\right)\left(1-{PE}_{3}\right)\dots\:(1-{PE}_{k})$$

$$\:{PD}_{c}=1-\left(1-{PD}_{1}\right)\left(1-{PD}_{2}\right)\left(1-{PD}_{3}\right)\dots\:(1-{PD}_{k})$$

### Genetic distances

When more than one population is analyzed, the Foren-STR software estimates the genetic distances between them using two approaches. The first is the standard Nei D_A_ distance, which has been successfully used with microsatellite data [9]. The Nei D_A_ distance is calculated as follows:

$$\:{D}_{a}=1-\frac{1}{r}{\sum\:}_{j}^{r}{\sum\:}_{i}^{{m}_{j}}\sqrt{{x}_{ij}{y}_{ij}}$$

where *x*_*ij*_ and *y*_*ij*_ are the frequencies of allele *i* at locus *j* in each population, *m*_*j*_ is the number of alleles at locus *j*, and *r* is the number of analyzed loci.

The second method used to estimate genetic distances among populations using STRs is the F_st_ method proposed by [13], calculated as:

$$\:{F}_{st}=\frac{\left({J}_{x}-{J}_{y}\right)/2-{J}_{xy}}{1-\:{J}_{xy}}$$

Where *J* is the expected homozygosity for *x* and *y* populations.

Both values range from 0 to 1, with increasing values indicating greater genetic distance between populations. The results of these calculations are presented in an n×n matrix showing both D_A_ and F_st_ values obtained from pairwise population comparisons (Table statistics > Distances tab). As with all estimations, these tables can be downloaded as .TXT files or copy to clipboard.

## Comparison of Foren-STR results with other software

The results obtained with Foren-STR were compared with those estimated using the STRAF and Arlequin software [6–8]. The latter was used for the comparison of the Hardy-Weinberg equilibrium (HW) test between both software. The results obtained with Foren-STR were comparable to those from this software (data not shown).

## Limitations

Currently, Foren-STR is only available for autosomal STR data and cannot be used for SNPs, Indels, or X- and Y-chromosome data. This software is designed to quickly perform calculations of forensic parameters that are useful for autosomal STR population analyses, either for publication purposes or case studies, without exploring population structure in detail. This is important to consider if the user wishes to explore their data in depth, as additional software may be required. However, all suggestions for improvement are welcome, as they are essential for enhancing the application.

## Conclusion

The current software is an alternative tool for quickly estimating forensic parameters and performing population genetic analysis using autosomal STR data, with reliable results. No bioinformatics training is required to analyze autosomal STR data with this web-based tool, as it is designed to be user-friendly. The results generated by Foren-STR are suitable for population data analysis, enabling identifications and kinship analysis using autosomal STRs, as well as for population data publications. Also, this software makes such analyses accessible, including to Spanish-speaking users.

## Electronic supplementary material

Below is the link to the electronic supplementary material.

Supplementary Material 1

## Data Availability

The code and example data are available in the repository at https://github.com/ENACIF/Foren-STR For any feedback regarding Foren-STR, please contact: mguardado@enacif.unam.mx.
